# Can telemedicine initiative be an effective intervention strategy for improving treatment compliance for pediatric HIV patients: Evidences on costs and improvement in treatment compliance from Maharashtra, India

**DOI:** 10.1371/journal.pone.0223303

**Published:** 2019-10-08

**Authors:** Sarit Kumar Rout, Yashwant R. Gabhale, Ambarish Dutta, Sudha Balakrishnan, Mamatha M. Lala, Maninder Singh Setia, Khanindra Bhuyan, Mamta V. Manglani

**Affiliations:** 1 Indian Institute of Public Health, Bhubaneswar, Odisha, India; 2 Pediatric Centre of Excellence for HIV Care, Department of Pediatrics, LTM Medical College and General Hospital, Sion, Mumbai, India; 3 UNICEF India, New Delhi, India; 4 Karanam Consultancy, Mumbai, India; 5 UNICEF state office, Maharashtra, India; NPMS-HHC CIC / LSH&TM, UNITED KINGDOM

## Abstract

**Background:**

India has recently introduced telemedicine initiatives to enhance access to specialized care at a low cost for the pediatric HIV patients, who face multiple challenges due to growing disease burden and limited preparedness of the health system to address it. There are limited evidences on the cost-effectiveness of these interventions. This study was undertaken in Maharashtra, a province, located in the western region of the country, to inform policy regarding the effectiveness of this programme. The objective was to estimate the unit cost of ART services for pediatric HIV patients and examine the efficiency in the use of resource and treatment compliance resulting from telemedicine initiatives in pediatric HIV compared to usual ART services.

**Methods:**

We selected 6 ART centers (3 from linked centers linked to Pediatric HIV Centre of Excellence (PCoE) and 3 from non-linked centers) randomly from three high, middle and low ART centers, categorized on the basis of case load in each arm. A bottom up costing methodology was adopted to understand the unit cost of services. Loss to follow up and timeliness of the visits were compared between the two arms and were linked to the cost.

**Results:**

The average cost per-visit was INR 1803 in the linked centers and that for the non-linked centers was INR 3412. There has been 5 percentage point improvement in lost to follow-up in the linked centers compared to non-linked centers against a back-drop of a reduction in per-pediatric patient cost of INR 557. The linkage has resulted in increase in timeliness of the visits in linked centers compared to non-linked centers.

**Discussion and conclusion:**

The telemedicine linkage led to an increase in the case load leading to a decrease in cost. The evidence on efficiency in the use of resource and improvement in treatment compliance as suggested by this study could be used to scale up this initiative.

## Introduction

India has the third largest Human Immunodeficiency Virus (HIV) epidemic in the world [1]. Approximately, 2.1 million” Indian live with HIV, the prevalence of the infection being 0.2% in the 15–49 age group, as on 2017, as per the UNAIDS report [2]. Currently, the number of Indians dying of Acquired Immunodeficiency Syndrome (AIDS) and related illnesses stand at 69,000 annually in 2017 and the deaths in the age group of 0–14 years is 2600. According to the same report, the death among the male in the age group of 15+ is much higher than the female counterparts in the same group. The condition can be economically disastrous and socially unjust for the children who are infected with HIV, especially for those whose parents die in an early age infected by the deadly virus. A sizeable number of children continue to become infected and die of HIV/AIDS [3] making pediatric HIV a unique burden to the healthcare system as well the Indian society at large. In India, there are approximately 61,000 children (less than 15 years old) living with HIV infection currently [2], and the number of AIDS orphans (0–17 years) stands at 930,000. The WHO [4] reinforces the need to reach children as early as possible and initiate the anti-retroviral treatment (ART) for all HIV positive children aged less than five years [3]. Although provisioning of ART has been in place throughout the country, the access to treatment is often unacceptably low [3]. This low access may be partially attributable to the dismal socio-economic condition of the households of the children living with HIV, aggravated by their parents’ suffering or demise in many cases.

In order to combat the problem of low access to appropriate treatment, many approaches including telemedicine have been introduced in most of the developed and developing countries of the world. Telemedicine holds great potential to improve the clinical treatment of a disease by enhancing access and quality of the health care service(s) [5]. Increasing penetration of broadband communication and interface device(s) has started effectively removing the cost and availability of this technology as a barrier to the uptake of telemedicine [1]. However, as the use of telemedicine pertaining to the health sector in the developed world has been well-established, it is still evolving in the developing world. For instance, in Britain, telemedicine is proposed as a cost-effective means of responding to structural problems in the organization of the NHS [6,7]. In the United States, telemedicine systems have been used, especially in rural areas, as a means of easing the problem of obtaining specialist advice and making referrals over wide distances [8,9]. The use of telemedicine has also been introduced in HIV-AIDS treatment, where adherence to treatment is essential for a comparatively healthy and longer life [10]. Such service deliveries have already been shown to be associated with favorable outcomes- virologic suppression and higher CD4 counts [11]. Other studies indicated these services are also associated with improved access, shorter visiting times, and higher patient satisfaction [12]. Furthermore, it is also observed that primary care physicians who access telemedicine services reported higher confidence in care and management of HIV infected cases [13,14]. A recent evaluation of a virtual support for pediatric HIV treatment found it to be extremely useful for decisions in complex cases [15]. Another recent study found that use of telemedicine has led to increased patient satisfaction, increased acceptability and found to be economical as observed by patients [16].

India has recently introduced telemedicine initiative to enhance access to specialized care at a low cost and moreover, it has also used this programme to facilitate HIV-AIDS management in certain states. The economic dimension pertaining to the cost and usefulness of the telemedicine-based intervention for care of people living with HIV-AIDS (PLHA) is critical to inform policy in a resource poor setting like India, where the health system faces multiple challenges to increase access to care for the most of the people staying in remote parts of the country. However, there is hardly any evidence on HIV infected individuals the cost effectiveness of telemedicine initiatives in the case of HIV in India. There are several evidences available in developed countries as well as South Africa regarding cost of ART services and its effectiveness. One study examining the cost, and cost-effectiveness associated with different strategies for using ART in India observed that antiretroviral therapy will lead to major survival benefits and is cost-effective by World Health Organization criteria. The same study reported availability of second-line regimens will further increase survival, but their cost-effectiveness depends on their relative cost compared with first-line regimens [17]. Another study shows the point estimate for the cost per DALY saved from the averted HIV infections for all interventions was much lower than the per capita gross domestic product in Indian states[18]. Similarly, another study also estimated the cost of HIV prevention intervention for commercial sex workers and suggested the cost varied depending upon the scale (coverage and service volume indicators). That study also pointed out the importance of scale specific cost information for planning[19]. Increase in the uptake and acceptance of telemedicine initiatives was reported by one study, which also pointed out that web-enabled telemedicine system can help in easy flow and better delivery of health care consultation to patients in remote areas [20]. There are other studies which generated evidence on cost effectiveness of mass media campaigns for HIV transmission and voluntary counselling to sex workers [21,22]. Furthermore, in a review paper, it was mentioned that though cost effectiveness studies assume importance, none of the studies done in low and middle income countries have complete cost data for a full range of HIV/AIDS prevention programmes [23]. This, however, is needed in order to generate empiric evidence with regards to widening the scope and reach of such interventions so that all PLHA receive quality care. Further, resource constraints especially, for the health sector in India is another compelling reason for conducting an economic evaluation which informs policy regarding the scalability of such initiative.

Therefore, a study was planned in Maharashtra with the following objectives:

1. To estimate the unit of cost of ART services in telemedicine linked and non-linked centers in Maharashtra.
2. To examine and compare efficiency in the use of resource and treatment compliance resulting from telemedicine initiatives in pediatric HIV compared to usual ART services to provide useful information for scaling up these initiatives

## Materials and methods

### Study setting

In October 2013, Pediatric HIV Telemedicine Initiative, e-decentralized model of expert Pediatric HIV care and referral services was established in Maharashtra, as a multi partner collaboration of the National AIDS Control Organization’s Pediatric HIV Center of Excellence at Sion Hospital, Mumbai, Maharashtra State AIDS Control Society (MSACS), National Health Mission in Maharashtra, Municipal Corporation of Greater Mumbai (MCGM) and UNICEF. The state has a high burden of HIV (Adult HIV Prevalence 0.42%, PLHIVs-315,849, CLHIVs-26,807, Children on ART-13,913). The telemedicine initiative was a video-linked delivery of expert pediatric services, designed to reach the unreached children with quality care. The services included expert opinion and guidance of pediatric HIV ART initiation, nutrition and adherence counseling, review for the first line failure, pediatric HIV mortality reviews and also capacity building for health personnel. For this study, we identified ART centers which are linked to PCoE and non-linked centers selected on the basis of sampling design mentioned below to compare the cost and efficiency of the intervention.

Currently, Maharashtra has 86 ART Centers—16% in Mumbai and 84% are in the rest of the state. About 50% of ART centers in 33 districts of Maharashtra (excluding Mumbai) are linked to telemedicine. In all, 190 telemedicine sessions were completed till 30-June-2015, including 382 clinical case discussions, three protocol discussions, 78 one-to-one counselling sessions, 57 death reviews, and 50 counseling training sessions. All medical officers in the telemedicine linked centers have had at least four rounds of mentoring sessions. In each teleconferencing session, the ART centers discussed at least one pediatric case—the discussion included clinical management, counselling, nutritional information, and any other issue relevant to the case.

### Sampling

We used a multi-stage sampling strategy for selection of ART centers for this study.

#### Stage 1: Selection of the ART centers for inclusion in the study

We included only those ART centers that were operational for the entire period from October 2013 through August 2015. There are 72 ART centers in Maharashtra; of these 35 are linked to the PCoE by telemedicine facility (referred to as linked centers) and 37 are not linked to the PCoE (referred to as non-linked centers). Among these centers 60 (32 linked and 28 non- linked) were eligible for inclusion in the study. From 72 ART centers, 60 ART centers (28 Non-linked and 32 linked) were found to be operational throughout the said period and were included in the analysis. For the linked centers additional selection criteria were placed. A center was included as a Linked center if they had at least five regular video conference sessions. The median (range) of the number of regular video-conference sessions in these centers were 12 (3–37). One Linked ART center had only three regular video conferencing session (less than the minimum required to be included in the Linked group). The expert services provided in a video conference session included real time consultation with the patients & their caretakers along with the team of doctors and counselors treating them at the peripheral level, in order to provide them expert guidance on various aspects of HIV management-HIV diagnosis, ART initiation, early recognition, treatment and/or prophylaxis of opportunistic infections, drug toxicities, treatment failure, nutrition and adherence counseling.

#### Stage 2: Selection of six ART centers for clinical data abstraction

On this basis, 59 ART centers from both the categories (28 non-linked and 31 linked) were selected for this study. All the 59 linked and non-linked ART centers were divided into tertiles according to the case load of the total number of registered patients in the ART centers from October 2013 through August 2015. The lowest to highest tertile was classified as low, moderate, and high case load respectively. Among the 31 linked ART centers, 11 were classified as low case load centers, 10 as moderate case load centers, and 10 as high case load centers. Among the non-linked ART centers, 10 were classified as low case load, 9 as moderate case load, and 9 as high case load. We selected one center from each tertile randomly (using computer programme) for our study. Thus, at the end of this sampling strategy, we had three linked ART centers and three non-linked ART centers (one each in the low, moderate, and high case-load). The three linked ART centers were: 1) Bhandara (Ever Registered- 574); 2) Jalna (Ever Registered- 771); and 3) Ahmednagar (Ever Registered- 1967). The three non-linked centers were; 1) Akola GMC (Ever Registered- 747); 2) Pandharpur (Ever Registered- 1515); and 3) Kolhapur CPR (Ever Registered- 1669).

### Costing methodology

This costing exercise was undertaken using a provider perspective to estimate a unit of service provided though PCoE-linked and non-linked centers. A bottom-up costing approach (micro-costing) was used to estimate the unit cost of services in the ART centers to achieve more accurate and scientific results. In a bottom up costing methodology, each activity undertaken to produce output in a given time period is identified and relevant cost for that activity is estimated. In a complex health service provision, where multiple actors are involved in the provision of services, accuracy in cost estimation can be obtained when all the activities are accurately identified and costed. For instance, the human resource or administrative branch in a hospital has multiple functions and when we are undertaking costing exercise for a particular activity (for instance outpatient care), appropriate criteria need to be developed for assigning their contribution for this particular function. Here, the provision of ART services includes complex organizational structure where resources need to be apportioned for administrative and other overhead costs which can be more accurately dealt-with using a bottom-up approach. Further, the cost data was collected from a provider perspective showing the costs incurred for providing ART services were included in the analysis. In the first step, the services were identified for the ART center namely- out-patient services (direct cost centers), counseling, pharmacy and diagnostic services (intermediate cost center) and indirect cost center (providing non-medical support such as administrative, laundry and catering services). Secondly, various resources/inputs were identified for each service and costs were allocated for each cost center based upon the resources used. Appropriate allocation criteria was used to apportion the expenditure of intermediate and indirect cost centers to the patient cost centers.

We estimate the unit cost of ART service for any patient and pediatric patient separately. In this study, the pediatric population was defined as those under the age of 18 years of age. We estimated the cost of each item separately including human resources, administrative cost and capital cost for each of the ART centers. For obtaining the cost on pediatric HIV patients in an ART center, we applied the ratio of pediatric to total HIV population in the ART center with each of the cost item. This was done because, the cost of the pediatric patients was not mentioned separately and both pediatric and adult patients consume the resources provided to the ART center. The apportioning factor used here was the ratio of pediatric population to total HIV population in a particular center.

For capital items (CD 4 machines, computer, furniture and other items), we annualized cost using following formula [24–26]:

$$AV=RC*[\frac{r{\left[r(1+r)\right]}^{n}}{[{\left(1+r\right)}^{n}]-1}]$$

Where, AV = Annualized Value, RC = Replacement Cost, r = rate of discount (here 3%), and n = average useful life years.

The annualized cost of each item was multiplied with the respective quantity for each item and then this was apportioned to the proportion of pediatric population to find out the cost of pediatric patients. The cost of each individual item thus obtained was then added up to calculate the total cost of capital item of each ART.

### Overhead cost

For hospital building, the monthly rent per hundred square meters per month for the location where the respective ART center situated, were collected. The rent per month was calculated and were then multiplied with 12 to get the annual rent. The obtained amount was apportioned to the ratio of pediatric to total HIV patients. The annual cost of electricity, water, internet, telephone and laundry for each ART center were also apportioned to the pediatric population in the respective ART centers.

## Data collection and analysis

The cost data was collected for the financial year 2014–15 using a structured questionnaire indicating different cost variables from the 6 ART centers. The questionnaire was developed as per the ART functions defined in the NACO guidelines. The cost data was collected using a standardized costing tool and included different items- salary, equipment, medicine, office stationary, electricity, telephone, water fees, expenditure on administration, training, travel and capital items from the six ART centers.

### Treatment compliance

In this analysis, we defined treatment compliance based upon two parameters: loss to follow up and timeliness of visits. All the pediatric patients registered at the 6 ART centers between October 2013 to August 2015 (23 months) were included for this analysis. Their visits to the ART centers for consultation, which may be routine or for certain perceived or real complications, were recorded in their respective patient cards. We extracted the dates of these visits and calculated the time interval between two consecutive visits. Thus for every patient, the time-interval between the consecutive visits to the ART centers (hereinafter referred to as time-interval only) were extracted. The time-interval data was unbalanced, as the patients were registered during the study period in a staggered fashion. So some patients could have made 23 (and even more) visits during the study period, whereas those registered in later part of this period could not have made as many visits. However, the data was arranged as panel data, whereby the repeat measures of time-intervals, depending on the number of visits made by those patients, were analyzed.

Further, the study period- 1^st^ of October 2013 till 31^st^ of August 2015—was categorized into three groups namely–“earlier”: first eight months of intervention; “intermediate”: the following eight months and “later”: the later seven months. This was done with a view to stratify the entire study period, as we expected variation in the “telemedicine linkage effects” of PCOE in these three phases, due to difference in the “maturity” as well as the “fatigue” of the linkage.

Time-interval variable was treated as a continuous variable and its median with inter-quartile range as well as mean and standard deviations were used to summarize and compare between PCOE-linked and non-linked ART performance. The time-interval variable was then further dichotomized into “delay and without-delay” groups based on whether the time-interval was more than and equal to 32 days or not, assuming ART drugs were dispensed for 30 days during each visit for the patient at the ART center.

Extreme outliers of time-intervals, as defined by gaps of more than 180 days, which were very few, were excluded from the analysis. Loss to follow-up pediatric patients was also considered for this study and this was compared with the unit of ART services at both arms.

Time-intervals were compared between PCOE-linked and non-linked centers using parametric t-test and also non-parametric Man Whitney U test. Proportions of patients attending ART centers without-delay were also compared and their statistical significance tested using chi-square test.

### Efficiency in use of resource

The efficiency parameter shows whether the same output can be attained with less inputs or resource or output can be maximized with less inputs. Here, the efficiency in the use of resources is defined in terms of cost per visit in the linked centers compared with the non -linked centers. After having estimated the costs of the six sampled ART centers (for all patients and pediatric patients), we compared the PCOE-linked ART centers to their non-linked counterparts, with regards to the visits made by the pediatric HIV clients using linear regression framework. This was to estimate the difference in per visit cost of linked vs non-linked ART center for pediatric patients. The dependent variable for this analysis was cost/visit of pediatric patients and the independent variables were their linkage status. Since it could be argued that the linked and non-linked centers had different patient load and therefore difference in patient visits historically, the regression framework offered us the opportunity to control for the historical patient load in the model and estimate the adjusted cost-difference.

$$Y=\beta_{0}+\beta_{1}X_{1}+\beta_{2}X_{2}$$

The coefficient that is being primarily studied is β_1_.

### Treatment compliance and efficiency in resource use

In order to show whether telemedicine has led to efficient use of resources with an improvement in treatment compliance, we presented a comparative picture of the efficiency in the use of resources and treatment compliance defined as improvement in the lost to follow-up between PCoE-linked and non -linked centers. non-linked The cost per-patient was estimated by dividing the total cost with the ever-registered pediatric patients in the respective districts. The average per-patient cost for the linked and non-linked centers were calculated separately and the differences between the two were defined as the efficiency in the use of resources if the linked center has resulted in decline in cost per patient compared with the non-linked center.

Lost to follow-up was defined as per the NACO guideline, that is any person failing to access the ART services for three consecutive months after the first visit. The percentage difference between the lost to follow-up cases in the linked and non-linked centers was used in the analysis.

## Results

### a. Cost of ART visit

The overall annual delivery cost of anti-retroviral therapy (ART) in the telemedicine-linked centers was INR 7,04,40,014 and that in the telemedicine-non-linked centers was INR 7,76,53,940. The overall per-visit cost per ART center was INR 2745.6 (US$ 41). The per-visit cost in the telemedicine-linked ART center on an average was INR 1898.2 (US$ 29) and same in the centers not linked to telemedicine was INR 3592.9 (US$ 54). However, there were wide differences in the per-visit cost across ART centers varying from INR 734 in Ahmednagar to INR 3551 in Jalna in the linked districts and from INR 719 in Kolhapur to INR 5661 in Pandharpur in the non-linked districts. The wide variations in the average cost across the districts were due to the uneven distribution of visits. For instance, in Jalna total 1500 visits were reported in comparison to 58393 in Ahmednagar. The efficiency in the delivery of services in terms of cost by Ahmednagar was completely negated by the low patient attendance in Jalna (Table 1).

**Table 1:** Per-visit ART delivery cost for any patient (in INR).

District	Human Resource	Consumables	Equipment	Medicines	Overhead	Total cost	Per-visit cost	Average visit cost
**Linked**								1898
Ahmednagar	3629931	51949	224029	37547859	1522150	42975918	734	
Jalna	1679815	33299	307003	3050411	338004	5408532	3551	
Bhandara	3052270	26225	259981	18704436	314500	22357412	1409	
**Non-linked**								3593
Kolhapur	3635511	31811	145864	23333799	493530	27640514	719	
Akola	2655776	22009	284183	16598107	307331	19867406	4399	
Pandharpur	2878143	52324	210145	25750630	1445208	30336449	5661	
**Total**	**17531446**	**217617**	**1431204**	**124985241**	**4420723**	**148586231**	**16474**	**2746**

The distribution of the total cost under four broad headings revealed that, the share of medicines was the major driver in determining the cost in the ART centers. The share of drugs constituted of more than 80% of the total cost apart from Jalna where it was 57%. The share of human resources varied between 8% -13%; Jalna being an exception where the staff salaries comprised of 30% of the total share of the cost. The share of equipment and consumables was least yet consistent for all the centers (1%) except for Jalna (6%).

### b. Cost of pediatric HIV

With regards to the pediatric patients, the overall ART service delivery cost per-visit in the three telemedicine-linked center was INR 1,30,86,865 and the cost in the telemedicine-non-linked center was INR 1,53,82, 774. For the three linked centers, the average cost per-visit was INR 1803 (US$ 27.3) and that for the non-linked centers was INR 3412 (US$ 51.6); nearly two times the cost of the linked centers. In the linked centers the cost per-visit was less due to higher patient attendance showing efficiency in the delivery of ART services (Table 2).

**Table 2:** Per-visit ART delivery cost for pediatric patients (in INR).

District	Human Resource	Consumables	Equipment	Medicines	Overhead	Total cost	Per-visit cost	Average visit cost
**Linked**								1803
Ahmednagar	706119.95	11226.909	39948.543	6291513.2	266017.17	7314825.8	716.79	
Jalna	229374.98	5672.6	40443.819	220807.09	35000.4	531298.89	3541.99	
Bhandara	957639.93	9429.4636	64854.203	3995600.4	213215.18	5240739.2	1149.28	
**Non-linked**								
Kolhapur	459033.16	3997.3569	23248.353	4252716.2	62016.929	4801012	993.59	3412
Akola	1130254.4	9233.0439	58673.083	6553586.8	128929.1	7880676.4	4165.26	
Pandharpur	289504.51	5195.2908	20372.029	2242518.4	143495.83	2701086	5077.23	
Total	**3771926.9**	**44754.664**	**247540.03**	**23556742**	**848674.62**	**28469638**	**15644.15**	**2607**

### c. Cost-differences between PCOE-linked and non-linked centers

The results clearly demonstrate that the PCOE-linkage resulted in a cost savings of Rs 1610/visit in the PCOE-linked center as compared to non-linked centers. After adjustment for ever registered patients up to October 2013, representing the pre-linkage burden, the efficiency metrics improved to Rs 1878. Although the confidence intervals were reasonably wide with not significant p-values for the estimates, this is expected in a small sample, consisting of only 3 units in each arm. But, the results signify that the linkage had improved the pediatric patient attendance considerably, which resulted in reduction in per unit service-delivery cost (S1 Table).

### d. Efficiency in resource use and treatment compliance

The linking of per-pediatric patient cost with treatment compliance defined as difference between lost to follow-up cases in PCoE-linked and PCoE-non-linked showed a 5 percentage point improvement in lost to follow-up in the linked centers compared to non-linked centers against a back-drop of a reduction in per-pediatric patient cost of INR 557. As mentioned in the table the per-patient cost in the linked ART centers was INR 4513 as compared to INR 5069 in the PCoE-non-linked centers (Table 3).

**Table 3:** Cost per-pediatric patient in PCoE-linked and non-linked ART centers.

	District	Ever registered (n)	Total Cost (INR)	Cost per-patient (INR)	Average cost	Lost to follow-up (in %)
**Linked**	Ahmednagar	1967	7314826	3718.8	4513	4
	Jalna	771	531299	689.1		
	Bhandara	574	5240739	9130.2		
**Non-linked**	Kolhapur	1669	4801012	2876.6	5069	9
	Akola	747	7880676	10549.8		
	Pandharpur	1515	2701086	1782.9		
**Total**		**7243**	**28469639**	**28747.3**	**4791.2**	

### e. Post linkage changes in timeliness of treatment he pediatric HIV patients

**Fig 1:**
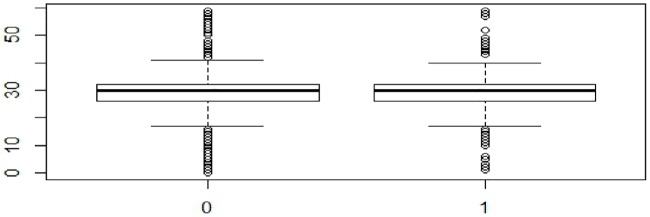
Comparison of median time-interval between PCoE-linked and PCoE-non-linked centres in the first 8 months.

**Fig 2:**
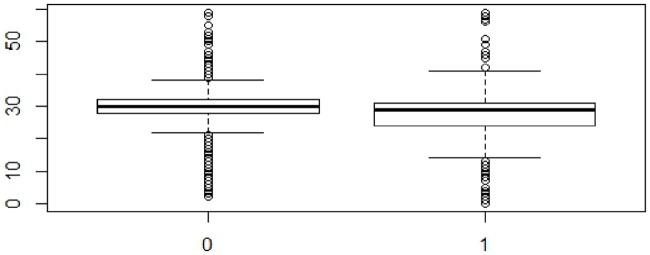
Comparison of median time-interval between PCoE-linked (1) and PCoE-non-linked centres in the middle 8 months.

**Fig 3:**
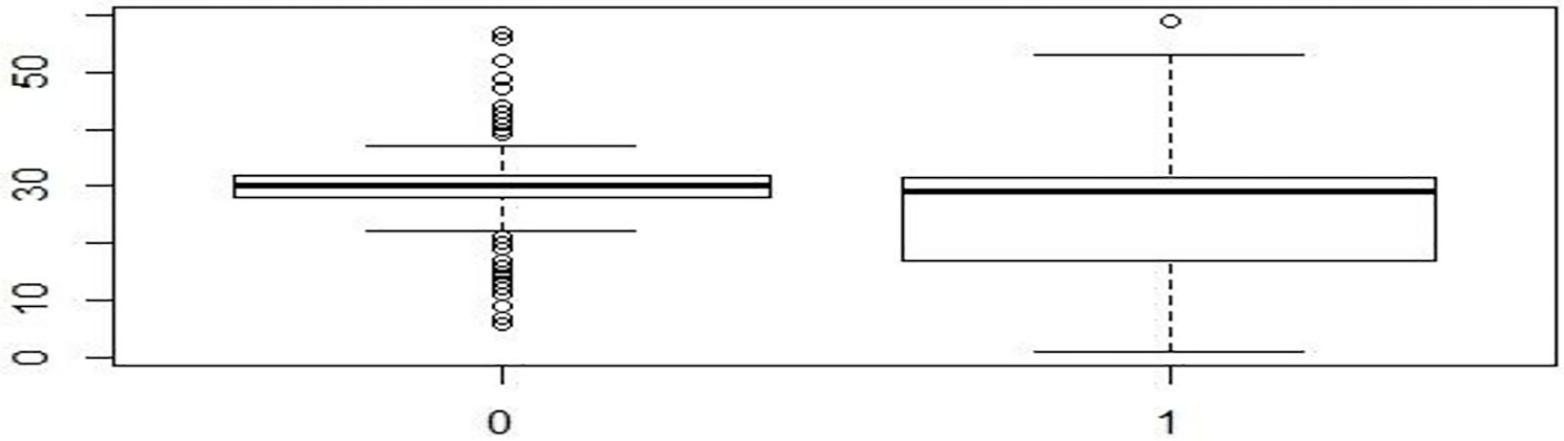
Comparison of median time-interval between PCOE-linked and PCOE-no-linked centres in the later 7 months.

The findings as presented in (Figs 1, 2 and 3) and Table 4 suggested that the average and median time-interval in the early phase were more for the pediatric patients in the PCoE-linked ART centers as compared to their counterparts in the non-linked centers. However, the difference was not statistically significant (mean and median time-intervals in PCoE-linked centers 33.2 and 31 respectively vs mean: 31.25 and median: 30 in the PCoE-non-linked centers, (p>0.05). This pattern changed in the intermediate phase of the telemedicine linkage initiative, when the average and median time-interval for children attending linked ART centers became smaller than that in the non-linked facilities (mean and median time-intervals in linked centers 30.43 and 29 respectively vs. mean: 31.01 and median: 30 in the non-linked centers, (p<0.05). This advantage that was evident in the PCoE-linked center was maintained in the later phase of the initiative also (mean and median time-intervals in linked centers were 29.82 and 29 respectively vs. mean: 31.20 and median: 30 in the non-linked centers, (p<0.05).

**Table 4:** Comparing the delay across various phases of the telemedicine initiative, between PCoE-linked and PCoE-non-linked ART centers.

		PCOE-linked ART centers	PCOE-non-linked ART Centers	p-value
**Earlier phase of telemedicine support**	Median	31	30	0.163
	Mean	33.2	31.25	
	% people with gaps<32 days (i.e. without delay)	59.7	64.5	0.12
**Intermediate phase of telemedicine support**	Median	29	30	0.01
	Mean	30.43	31.01	
	% people with gaps<32 days (i.e. without delay)	71.2	63.7	0.04
**later phase of telemedicine support**	Median	30	30	0.02
	Mean	29.82	31.2	
	% people with gaps<32 days (i.e. without delay)	65.9	68.5	0.5

The proportion of the visits made, without-delays (or time-interval between subsequent visits < 32 days), were less in the PCoE-linked ART centers, in the earlier phase; 59.7% in linked vs. 64.5% in non-linked centers. This trend reversed in the intermediate phase– 71.2% visits undertaken without-delay in the linked ARTs as compared to 63.7% in the non-linked centers, p<0.05. The proportions became almost equal in the later phase of the telemedicine initiative (Table 4).

## Discussion

This study has made a modest attempt to estimate the cost of ART service delivery for HIV patient, pediatric HIV patient separately in the PCoE-linked and non-linked centers in Maharashtra and examined whether the telemedicine initiative for pediatric HIV patients has led to improvement in treatment compliance by comparing cost with the reduction in the lost to follow-up cases. To our knowledge, this is the first ever study in India, which examined the cost of pediatric HIV patients and efficiency in the use of resources using a comprehensive costing methodology. This study presented several important evidences for policy uptake in the country especially for designing similar intervention strategies for children and other vulnerable population in other settings. The findings suggested that the average cost per-visit was INR 2746 (US$ 41) for any patient and that for the pediatric population was 2607 (US$ 39.5) in 2014–15. It is important to mention here that on average a patient visit once in a month to the ART center and the medicine is provided for the whole month and the per visit cost should be understood in this perspective. In the absence of large evidences available in India, it is difficult to compare this results with other studies. Whatever limited evidences available in India, are only related to specific interventions—voluntary counseling and testing centers [27] or cost per DALY averted [22]. Another study estimated the cost of provisioning of ART from program perspective and the cost was INR 668 per month [28]. Similarly, another study reported the average cost of client on ART was INR 1287, which did not take into account the capital cost [29].

Further, our study suggested that the telemedicine initiative for the HIV pediatric patients has led to improve efficiency in the use of resources by reducing the cost in the PCoE-linked centers in comparison to non-linked centers. Due to telemedicine implementation, the patient visits in the PCOE-linked centers has increased resulting in the decline of the per-visit cost. It was observed that the per-visit cost in the non-linked centers was nearly two times than that in the linked centers. The majority of the cost component was drugs followed by human resources.

The study also revealed that the telemedicine implementation resulted in a less cost per visit amounting to f INR 1610/ per visit in the linked centers compared to non-linked centers. However, when this was adjusted to ever registered patients, the cost efficiency further increased to INR 1878, per-visit. This is not statistically significant as the confidence intervals were large due to limited samples in both arms. However, the results signify that the telemedicine has led to reduction of cost due to increase in patient visits in the linked centers.

Another significant finding of this study was the improved efficiency of the program measured by comparing cost and treatment protocol defined as decrease in lost to follow-up cases where a 5 percentage point improvement in lost to follow-up was observed with a reduction of INR 557 per-pediatric patient cost in the linked centers compared to non-linked centers.

With regards to the treatment compliance, this study used the timeliness of visits examining interval between subsequent visits in the linked and non-linked centers and whether there has been a change in the pattern due to introduction of telemedicine. These findings clearly suggested that the timeliness of the visit increased in the linked ART centers compared to their counter parts. During the early phase of the intervention, the mean and median time interval between subsequent visit was a little higher in the ART linked centers compared with the non-linked centers. However, as the initiative matured over time, the effect of the telemedicine support from PCOE was tangible as the mean/median time-interval dropped in the linked ART centers as compared to their non-linked counterparts. Further, the proportion of children attending the linked ART centers without delay saw a substantial increase in contrast to non-linked ART centers.

This favorable change in the linked ART centers, experienced during the “intermediate phase”, which is perhaps attributable to telemedicine support from PCOE, was partially diluted during the last 7 months of the support initiative—the “later” phase. It was observed that—the median/mean time-interval was slightly better in the linked ART centers compared to non-linked centers, however, the proportion of visits without delays started declining. This suggested that the advantages gained could not be sustained during the last phase of the analysis. This could be due to several reasons: systems “fatigue” an important reason among them. This is an important finding, which demands designing innovative strategies to improve the effectiveness of the programme by sustaining the motivation and improving the skills of the medical and paramedical staff involved in this process.

### Strengths and limitations

The study did not use more commonly used outcomes of “cost effectiveness” studies—Disability Adjusted Life Years (DALYs) and death averted, because of limited availability of data in a mid-term evaluation, the objective of which was to generate evidence whether the intervention is in right direction and is achieving the intended results.

We used improvement in treatment compliance comprising up loss to follow up and timeliness of visits to show the effectiveness of the programme, as death data defined as health outcome, was limited in a small sample size. The sample size was small, 3 centers in both the arms, designed so, keeping in view the time and resource constraint in this mid-term evaluation framework of the project. This evaluation was undertaken to show whether the programme was implemented as per the design and whether it produced positive results. Moreover, according to the findings of the study, the cost differences between the linked and non-linked centers was large and this was compared with the loss to follow up to draw appropriate conclusions of this study. This small sample size led to both the difference estimates (adjusted and unadjusted) being not statistically significant, however the point estimates, that are the actual difference gives us some indication on the variation between the two types of centers. Despite these limitations, this study for the first time to our knowledge, shows the effectiveness of telemedicine initiatives for improving pediatric HIV outcomes by comparing cost and treatment compliance. These findings could be used to inform policy regarding the effectiveness of the programme and encourage research community to develop additional robust knowledge for scaling up the intervention to increase health care access in the remote part of the country with less cost as suggested by this study.

## Conclusion

This study for the first time generated evidence on the cost of pediatric ART delivery through telemedicine and linked this cost to treatment compliances in India. The telemedicine linkage initially led to an increase in the case load leading to a decrease in the cost of provisioning services. Although there was no uniformity in the case load among the linked centers overall, there was a positive impact of the telemedicine linkage. As evident, the cost per visit of ART service at the linked centers was less than that of the non-linked centers. Importantly, this initiative led to improvement in treatment compliance in the linked centers compared to non-linked centers. These advantages demonstrated through this initiative could be encouraged and scaled-up in similar settings.

## Supporting information

S1 Table Difference in average cost per visit of pediatric HIV patients between PCOE-linked and unlinked centers. (DOCX)

S1 File Costing data collection tools. (DOCX)

S2 File Data sets and analysis. (XLSX)
