# Supplementary material for: Can telemedicine initiative be an effective intervention strategy for improving treatment compliance for pediatric HIV patients: Evidences on costs and improvement in treatment compliance from Maharashtra, India
Source: PLoS One. 2019 Oct 8;14(10):e0223303. doi: 10.1371/journal.pone.0223303 (PMC6782091; doi:10.1371/journal.pone.0223303)
Supplement: S1 Table — (DOCX) [file pone.0223303.s001.docx]

**Supplementary Table 1. Difference in average cost per visit of pediatric HIV patients between PCOE-linked and unlinked centers**

|  | **Beta coefficient** | **95% Confidence Interval** | ***P* value** |
| --- | --- | --- | --- |
| **Unadjusted** | 1609 | -5823 to 2604 | 0.35 |
| **Adjusted*** | 1879 | -6963 to 3205 | 0.49 |

*adjusted for ever-registered patient
