# Supplementary material for: Can telemedicine initiative be an effective intervention strategy for improving treatment compliance for pediatric HIV patients: Evidences on costs and improvement in treatment compliance from Maharashtra, India
Source: PLoS One. 2019 Oct 8;14(10):e0223303. doi: 10.1371/journal.pone.0223303 (PMC6782091; doi:10.1371/journal.pone.0223303)
Supplement: S1 File — (DOCX) [file pone.0223303.s002.docx]

**Cost data collection tool**

Facility type: Antiretroviral Therapy centre

Information about the facility

| Interview Date | __/__/__ |
| --- | --- |
| State name |  |
| District Name |  |
| Facility Type |  |
| Facility Name |  |
| Name of the investigator |  |

This tool intends to collect information pertaining to following heads for the ***financial year 2014-15*:**

***Section 1: General Information***

1. Please tell me how many days per week this facility is closed? : __________ (Days per week)
2. Please tell me how many hours per day this facility is open? : _________ (Hours per day)
3. If the facility remains closed on Public holidays then mention total public holidays in last year: _________(Days in year)

**Section 2: Population covered under facility**

| Total HIVpopulation under the ART Centre | Total=  Male=  Female=  Children (under 0-5 years)=  Children (0-18 years)= |
| --- | --- |

**Section 3: Sources of Revenue**

|  |  | **Amount collected during the period of data collection (2014-15)** |
| --- | --- | --- |
|  |  |  |
|  |  |  |
|  |  |  |
|  |  |  |
|  |  |  |
|  |  |  |
|  |  |  |
|  |  |  |

**Section 4: Human resource-Salary and fringe benefits details**

| ***Staff No. Code**** | ***Job title^@^*** | ***Monthly gross salary***  ***(inclusive of all allowances or deductions)*** | ***Annual Incentive received for trainings***  ***(TA/DA received for trainings)*** | ***Period/days of posting in the year 2014-15^$^*** | ***Days of absence from this health facility in the period of posting in the year 2014-15^$$^*** | ***Time contribution towards administration (per working day)*** |
| --- | --- | --- | --- | --- | --- | --- |
|  |  |  |  |  |  |  |
|  |  |  |  |  |  |  |
|  |  |  |  |  |  |  |
|  |  |  |  |  |  |  |
|  |  |  |  |  |  |  |
|  |  |  |  |  |  |  |
|  |  |  |  |  |  |  |
|  |  |  |  |  |  |  |
|  |  |  |  |  |  |  |
|  |  |  |  |  |  |  |
|  |  |  |  |  |  |  |
|  |  |  |  |  |  |  |
|  |  |  |  |  |  |  |
|  |  |  |  |  |  |  |
|  |  |  |  |  |  |  |
|  |  |  |  |  |  |  |
|  |  |  |  |  |  |  |
|  |  |  |  |  |  |  |
|  |  |  |  |  |  |  |
|  |  |  |  |  |  |  |
|  |  |  |  |  |  |  |
|  |  |  |  |  |  |  |
|  |  |  |  |  |  |  |
|  |  |  |  |  |  |  |
|  |  |  |  |  |  |  |
|  |  |  |  |  |  |  |

CODE LIST: 1-SMO, 2- MO, 3 LT, 4 Counsellor, 5 Data Manager, 6 Staff Nurse, 7 Care Coordinator 8 Institute Nurse, 9 Pharmacist 10 Others

*^@^Add extra rows, if more perssonel, ^$^Include any person posted during 2014-15, but now transferred/not posted and include any person not posted at this facility during 2014-15, but providing services in ARTC for few days or week/ month/ year. ,^$$^Leave of any nature, training days, etc.*

**Section 5 : Details of Consumables. Material and Supplies consumed in the facility**

| ***Consumables. Material and Supplies*** | ***Quantity*** | ***Usage period in years*** | ***Price per unit*** |
| --- | --- | --- | --- |
| Patient pre ART screening register |  |  |  |
| Patient condom register |  |  |  |
| Patient enrollment register |  |  |  |
| Paediatric record register |  |  |  |
| Drug stock register |  |  |  |
| Paediatric drug stock register |  |  |  |
| Patient record cards |  |  |  |
| Paediatric record cards |  |  |  |
| Dispatch register |  |  |  |
| Attendance register |  |  |  |
| Rubber stamps |  |  |  |
| Stock register |  |  |  |
| Patient pre ART screening register |  |  |  |
| Patient condom register |  |  |  |
| Patient enrolment register |  |  |  |
| Paediatric record register |  |  |  |
| Drug stock register |  |  |  |
| Article indent book |  |  |  |
| Attendance register |  |  |  |
| Bath soap |  |  |  |
| Carbon paper |  |  |  |
| Cash receipt book |  |  |  |
| Disinfectant fluids (Phenly-Ltr) |  |  |  |
| Harpic |  |  |  |
| Nirma |  |  |  |
| OPD card |  |  |  |
| Out-station dak book |  |  |  |
| Pencil |  |  |  |
| Broom |  |  |  |
| Photostat paper |  |  |  |
| Pocha |  |  |  |
| Poly bags for biowaste |  |  |  |
| Savlon solution |  |  |  |
| Spirit |  |  |  |
| Stamp ink |  |  |  |
| Stamp pad |  |  |  |
| Toilet brush |  |  |  |
| Towels + dusters |  |  |  |
| A-4 paper |  |  |  |
| Vim powder |  |  |  |
| BMI charts |  |  |  |

***Section 6:*** *Capital items (non-medical)*

|  | *Quantity* | *Price(cost)* | *Average life years* | *Used by* |
| --- | --- | --- | --- | --- |
| Computer |  |  |  |  |
| Printer |  |  |  |  |
| Almirah/cup board  (Big steel) |  |  |  |  |
| Almirah/cup board  (Small steel) |  |  |  |  |
| Almirahs/cup board  (Small wooden) |  |  |  |  |
| Armless chairs |  |  |  |  |
| Bed side attendant chair |  |  |  |  |
| Bed side locker |  |  |  |  |
| Bed side Screen |  |  |  |  |
| Bed side table |  |  |  |  |
| Buckets |  |  |  |  |
| CFL tubes |  |  |  |  |
| Bulbs |  |  |  |  |
| Clock /watch |  |  |  |  |
| Coat rack |  |  |  |  |
| Curtain rods |  |  |  |  |
| Curtains |  |  |  |  |
| Dressing trolley |  |  |  |  |
| Drum with tap for storing water |  |  |  |  |
| Examination bed or table |  |  |  |  |
| Fans |  |  |  |  |
| Foot step |  |  |  |  |
| Hand washing basin |  |  |  |  |
| Large medicine cupboard |  |  |  |  |
| Large steel benches |  |  |  |  |
| Large wooden benches |  |  |  |  |
| Mattress |  |  |  |  |
| Medicine trolley |  |  |  |  |
| Metal chair |  |  |  |  |
| Metal file cabinet |  |  |  |  |
| Mugs |  |  |  |  |
| Plastic bin |  |  |  |  |
| Refrigerator |  |  |  |  |
| Rubber / plastic shutting |  |  |  |  |
| Side Wall mounted fan |  |  |  |  |
| Side wooden racks |  |  |  |  |
| Sink |  |  |  |  |
| Stool (steel ) |  |  |  |  |
| Stool (wooden) |  |  |  |  |
| Stretcher |  |  |  |  |
| Swab rack |  |  |  |  |
| Telephone |  |  |  |  |
| Three seater steel chairs |  |  |  |  |
| Water purifier |  |  |  |  |
| File cabinet |  |  |  |  |
| Television |  |  |  |  |
| Television trolley |  |  |  |  |
| DVD PLAYER |  |  |  |  |

1-SMO, 2- MO, 3 LT, 4 Counsellor, 5 Data Manager, 6 Staff Nurse, 7 Care Coordinator 8 Institue Nurse, 9 Pharmacist 10 Others

***Section 7*** *Capital Items Medical*

|  | *Quantity* | *Price(cost)* | *Average life years* | *Annual maintenance cost* |
| --- | --- | --- | --- | --- |
| CD4 machine |  |  |  |  |
| Weighing machine, |  |  |  |  |
| Height measurement pole, |  |  |  |  |
| Blood pressure  (BP) apparatus, |  |  |  |  |
| Stethoscope, |  |  |  |  |
| Tuning fork |  |  |  |  |
| Hammer, |  |  |  |  |
| Torch, |  |  |  |  |
| Tongue depressor |  |  |  |  |
| Ophthalmoscope |  |  |  |  |
| Pulse oximeter |  |  |  |  |
| Digital camera |  |  |  |  |
| Lab equipment_1 |  |  |  |  |
| Lab equipment_2 |  |  |  |  |
| Lab equipment_3 |  |  |  |  |
|  |  |  |  |  |
|  |  |  |  |  |
|  |  |  |  |  |
|  |  |  |  |  |
|  |  |  |  |  |

***Section 8 Drugs- ARV***

| ***Name of drug*** | ***Quantity*** | ***Price per unit*** | ***Purpose of use***  ***(pediatric =1, adult=2, both=3)*** |
| --- | --- | --- | --- |
| Zidovudine + Lamivudine + Nevirapine |  |  |  |
| Stamivudine + L+N |  |  |  |
| Z + L |  |  |  |
| Stamivudine + L |  |  |  |
| Efavirenz |  |  |  |
| Tenofovir + L |  |  |  |
| Nevirapine |  |  |  |
| Atazanavir |  |  |  |
| Ritonavir |  |  |  |
| Lopinavir |  |  |  |
| Others |  |  |  |
| d4T6+3TC |  |  |  |
| d4T6+3TC + NVP |  |  |  |
| EFV 200 |  |  |  |
| EFV 50 |  |  |  |
| AZT60+3TC30 |  |  |  |
| AZT60+3TC30+ NVP 50 |  |  |  |
| ABC60+3TC30 |  |  |  |
| NVP 50 mg |  |  |  |
| NVP syrup |  |  |  |
| ddI 125 |  |  |  |
| ddI 200 |  |  |  |
| LPV/r 125 |  |  |  |
| LPV/r syrup |  |  |  |
| Cotrimoxazole DS |  |  |  |
| Cotrimoxazole SS |  |  |  |
| Cotrimoxazole suspension |  |  |  |
| Tenofovir + L + E |  |  |  |
| ABC600+3TC300 |  |  |  |
| *Drugs distributed to TB patients* |  |  |  |
| Isoniazid |  |  |  |
| Rifampicin |  |  |  |
| Ethambutol |  |  |  |
| Pyrazinamide |  |  |  |
| Streptomycin |  |  |  |
|  |  |  |  |
|  |  |  |  |

***Section 8 drugs- Other than ARV***

| ***Name of drug*** | ***Quantity*** | ***Price per unit*** | ***Purpose of use***  ***(pediatric =1, adult=2, both=3)*** |
| --- | --- | --- | --- |
| Acivir cream |  |  |  |
| Cap. Augmentin |  |  |  |
| Cap. Itraconazole |  |  |  |
| Clotrimazole cream |  |  |  |
| Inj. Acyclovir 250 mg |  |  |  |
| Inj. Amphotericin B 50 mg |  |  |  |
| Inj. Cefotaxime 1 gm |  |  |  |
| Inj. Ceftriaxone 500 mg |  |  |  |
| Inj. Ceftriaxone 1 gm |  |  |  |
| Inj. Clindamycin 300 mg |  |  |  |
| Inj. Clindamycin 600 mg |  |  |  |
| Inj. Gancyclovir 500 mg |  |  |  |
| Inj. Netilmycin 100 mg |  |  |  |
| Syp. Cremafin |  |  |  |
| Tab Acyclovir DT 200 |  |  |  |
| Tab. Acyclovir DT 400 mg |  |  |  |
| Tab. Acyclovir DT 800 mg |  |  |  |
| Tab Augmentin DS 1000 |  |  |  |
| Tab Avil 25 mg |  |  |  |
| Tab Azithromycin |  |  |  |
| Tab Ciplox 500 mg |  |  |  |
| Tab Ciplox Tz |  |  |  |
| Tab Clindamycin 300 mg |  |  |  |
| Tab Fluconazole 100 mg |  |  |  |
| Tab Fluconazole 200 mg |  |  |  |
| Tab Fluconazole 400 mg |  |  |  |
| Tab Gancyclovir 250/500 mg |  |  |  |
| Tab Metrogyl 400 mg |  |  |  |
| Tab Secnidazole 1 g |  |  |  |
| Tab Septran |  |  |  |
| Septran SS |  |  |  |
| Calamine lotion |  |  |  |
| Tab Clarithromycin |  |  |  |
| Inj. Fluconazole 200 mg |  |  |  |
|  |  |  |  |
|  |  |  |  |
|  |  |  |  |
|  |  |  |  |
|  |  |  |  |

**Section 9: Details of the Physical infrastructure**

| ***Particulars*** | ***Specify*** |
| --- | --- |
| Area of the building (Total hospital area in Sq. ft.) (Covered space) |  |
| Area of the building (Total ARTC area in Sq. ft.) (Covered space) |  |
| Area of the building (Total Hospital area in Sq. ft.) (Open space) |  |
| Area of the building (Total ARTC area in Sq. ft.) (Open space) |  |
| What is the rental price of 100 sq ft place where this centre is located? |  |
| Was there any expense on renovation or construction of accessory items during the period 2014-15 |  |

**Section 10: Utilities**

|  | ***Quantity*** | ***Expenditure*** | ***Utility*** | ***List services for which it is used.*** |
| --- | --- | --- | --- | --- |
| **Building** |  |  |  |  |
| Electricity |  |  |  |  |
| Water |  |  |  |  |
| Maintenance |  |  |  |  |
| Telephone |  |  |  |  |
| Internet |  |  |  |  |
| **Laundry** |  |  |  |  |
| **Others….** |  |  |  |  |
|  |  |  |  |  |
|  |  |  |  |  |

**Section 11: Annual services delivered**

| ***Codes*** | ***Services delivered*** | ***No of cases during 2014-15*** | ***No of children (0-5 years ) during 2014-15*** | ***No of children (0-18 years ) during 2014-15*** |
| --- | --- | --- | --- | --- |
|  | *Curative/OP patients* |  |  |  |
|  | *Counselling* |  |  |  |
|  | *Registration* |  |  |  |
|  | *Blood sample drawn* |  |  |  |
|  | ***No of laboratory tests done*** |  |  |  |
|  | CD4 done |  |  |  |
|  | Drug distribution to on ART |  |  |  |
|  | Others |  |  |  |
|  |  |  |  |  |
|  |  |  |  |  |
